# Riverine antibiotic resistome along an anthropogenic gradient

**DOI:** 10.3389/fmicb.2025.1516033

**Published:** 2025-02-26

**Authors:** Gangan Wang, Sarah Haenelt, Felipe Borim Corrêa, Ulisses Nunes da Rocha, Florin Musat, Junya Zhang, Jochen A. Müller, Niculina Musat

**Affiliations:** ^1^Department of Technical Biogeochemistry, Helmholtz Centre for Environmental Research, Leipzig, Germany; ^2^Department of Environmental Microbiology, Helmholtz Centre for Environmental Research, Leipzig, Germany; ^3^Department of Biology, Section for Microbiology, Aarhus University, Aarhus, Denmark; ^4^State Key Joint Laboratory of Environmental Simulation and Pollution Control, Research Center for Eco-Environmental Sciences, Chinese Academy of Sciences, Beijing, China; ^5^Karlsruhe Institute of Technology, Institute for Biological Interfaces (IBG 5), Eggenstein-Leopoldshafen, Germany

**Keywords:** riverine system, antibiotic resistome, metagenomic sequencing, anthropogenic activities, fluorescence *in situ* hybridization

## Abstract

The introduction of antibiotic-resistant bacteria into riverine systems through the discharge of wastewater treatment plant (WWTP) effluent and agricultural waste poses significant health risks. Even when not pathogenic, these bacteria can act as reservoirs for antibiotic resistance genes (ARGs), transferring them to pathogens that infect humans and animals. In this study, we used fluorescence *in situ* hybridization, qPCR, and metagenomics to investigate how anthropogenic activities affect microbial abundance and the resistome along the Holtemme River, a small river in Germany, from near-pristine to human-impacted sites. Our results showed higher bacterial abundance, a greater absolute and relative abundance of ARGs, and a more diverse ARG profile at the impacted sites. Overall, the ARG profiles at these sites reflected antibiotic usage in Germany, with genes conferring resistance to drug classes such as beta-lactams, aminoglycosides, folate biosynthesis inhibitors, and tetracyclines. There were also variations in the ARG profiles of the impacted sites. Notably, there was a high abundance of the oxacillin resistance gene *OXA-4* at the downstream site in the river. In the metagenome assembly, this gene was associated with a contig homologous to small plasmids previously identified in members of the *Thiotrichaceae*. The likely in-situ host of the putative plasmid was a close relative of *Thiolinea* (also known as *Thiothrix*) *eikelboomii*, a prominent member of WWTP microbiomes worldwide. Our results show that the effluent from WWTPs can introduce bacteria into the environment that act as shuttle systems for clinically relevant ARG.

## 1 Introduction

Antimicrobial resistance (AMR) undermines the effectiveness of antibiotics and other antimicrobial treatments, leading to increased morbidity and mortality from infections that were previously treatable. According to recent findings, an estimated 4.7 million deaths were associated with bacterial AMR in 2021 alone, and annual mortality could increase substantially without improved measures to curb the spread of AMR (Naghavi et al., 2024). Antibiotic-resistant bacteria (ARB) and antibiotic-resistance genes (ARGs) can circulate through various pathways in the human population but also via animals and the environment (Forsberg et al., 2012; Woolhouse et al., 2015; Martínez, 2019). For example, contaminated drinking and recreational water can contribute significantly to the contagion of pathogenic ARB, depending on regional socioeconomics (Collignon et al., 2018). The emission of ARB into the environment may also result in the transfer of their ARGs to environmental bacteria, e.g., via mobile genetic elements such as plasmids, transposons, and integrons, creating a gene pool with the potential for further horizontal dissemination to known and emerging pathogens (Sáenz et al., 2001; Czekalski et al., 2012; O’Flaherty et al., 2018, 2019; Amarasiri et al., 2020). In addition to being an arena for the evolution of novel ARG-host combinations, the environment can be viewed as a principal source of clinically relevant ARGs, given that resistance mechanisms predate the human use of antibiotics (D'Costa et al., 2011). The inextricable link between public health, animals, and the environment in the spread and evolution of AMR has been formulated in the *One Health* concept (Velazquez-Meza et al., 2022).

Riverine systems flowing through cities and other areas impacted by anthropogenic activities receive ARB and ARGs and thus play a role in the dissemination of AMR (Berendonk et al., 2015; Caucci et al. 2016; Rowe et al., 2016; Suzuki et al., 2017; Sivalingam et al., 2024). Many studies tracked ARBs and ARGs resulting from untreated sewage and effluent from WWTPs, which are usually not designed to remove bacteria (Lapara et al., 2011; Chen and Zhang, 2013; Marti et al., 2013; Sabri et al., 2020; Haenelt et al., 2023b; Kunhikannan et al., 2021). Monitoring of AMR in river water and other environments is often carried out by enumerating copy numbers of indicator genes by quantitative PCR (qPCR), such as *sul1*, *intI1*, and *tetA*, which are closely related to anthropogenic activities, and *bla_CTX-M_* and *vanA*, which are considered clinically relevant (Bradford, 2001; Koczura et al., 2016; Lee et al., 2021; Keenum et al., 2022; Gillings et al., 2015; Gunjyal et al., 2023; Haenelt et al., 2023a,b; Pruden et al., 2012). However, these indicator genes may not always reflect *in situ* AMR levels, as antibiotic use varies across countries and regions, causing discrepancies between indicator abundance and specific ARG levels at sampling locations (Prieto Riquelme et al., 2022). This analytical limitation can be addressed with metagenomic sequencing, an important tool for identifying known and novel ARGs and, to some extent, their hosts and associated mobile genetic elements (MGEs) in various samples (D’Costa et al., 2006). It helps to define the antibiotic resistome in a microbial community, i.e., the collection of ARGs present in clinically relevant pathogens but also in environmental bacteria that may act as reservoirs and sources of canonical and novel resistance traits (Wright, 2007; Martínez, 2008). It can provide valuable data for identifying regional indicator ARGs for qPCR-based analyses and other approaches. Consequently, metagenomic sequencing is widely used to monitor AMR in various environmental matrices, including riverine systems (Bing et al., 2015; Ma et al., 2016; Tang et al., 2016; Lekunberri et al., 2018; Di Cesare et al., 2023; Tarek and Garner, 2023).

In this study, we selected the Holtemme River as a model system to analyze the impact of anthropogenic activities on the resistome. The Holtemme River is a medium-sized watercourse formed by the convergence of second-order streams located in Saxony-Anhalt, Germany. Over a total length of 47 km, it flows from the Harz National Park to the Bode River. Land use along the Holtemme River ranges from a near-pristine region upstream to areas with high wastewater input [approximately 30% (v/v)] and increasing agricultural activity downstream (Wollschläger et al., 2017; Weitere et al., 2021; Schmitz et al., 2022; Haenelt et al., 2023a,b). In previous investigations at near-pristine and WWTP-impacted sites of the Holtemme River (Haenelt et al., 2023a,b), we enumerated the marker ARGs, *sul1* and *sul2*, and the gene encoding the class 1 integron integrase, *intI1*. These studies demonstrated that WWTP discharge was the principal source of these genes along the river sites, showing an abundance pattern that corresponded well with that of other micropollutants in the Holtemme River (Krauss et al., 2019; Beckers et al., 2020; Švara et al., 2021; Haenelt et al., 2023a,b). Furthermore, profiling microbial communities, combined with mathematical modeling, revealed that downstream communities only partially recovered to their near-pristine composition after WWTP discharge (Haenelt et al., 2023b).

Our current study focuses on the resistome along the Holtemme River from near-pristine to human-impacted sites, aiming at (i) investigating how anthropogenic activities impact bacterial abundance using single-cell fluorescence *in situ* hybridization and (ii) identifying and quantifying the changes in the microbial community and antibiotic resistome along with the increase of the human impact by a combination of qPCR and metagenomics.

## 2 Materials and methods

### 2.1 Sample collection and preparation

Surface water samples were collected in October 2020 after the local harvest from three sites along the land-use gradient of the Holtemme River (Supplementary Figure S1). The sites had been selected and sampled in previous studies that investigated the anthropogenic impact on the river (see references in the penultimate paragraph of the Introduction). Site 1 (N51°49′00.9″, E10°43′29.8″): Steinerne Renne is a near-pristine reference site about 2.3 km downstream of its spring [we refer to the site as “near-pristine” because it is located in the still unaltered stream bed and only traces of a limited number of anthropogenic pollutants, such as the insecticide diethyltoluamide, have been found (Weitere et al., 2021)]; Site 2 (Mahndorf, N51°53′06.2″, E10°57′47.2″) and Site 3 (Nienhagen, N51°56′29.7″, E11°09′31.1″) traverse urban and agricultural areas.

Site 2 is located approximately 8 km downstream from the Wernigerode-Silstedt wastewater treatment plant (with a physical capacity of 80,000 population equivalents) and is bordered on both sides by grassland. Site 3 is located approximately 8 km downstream of the WWTP of Halberstadt (physical capacity: 60,000 p.e.) and with agricultural fields adjacent to the sampling site (Weitere et al., 2021). One liter of surface water sample was collected from each sampling site and transported on ice in a thermostable box to the laboratory within 6 h. For each sampling site, volumes of 500 mL water samples were filtered through 0.2 μm pore size filters (Supor^®^200, Pall Corporation, NY, United States) for DNA extraction. Subsamples of 37.5 mL water were treated overnight at 4°C with 4% paraformaldehyde (electron microscopy grade; Electron Microscopy Sciences, PA, United States). Fixed samples were then filtered in triplicate on polycarbonate filters (GTTP02500 type; 25 mm diameter; Merck Millipore, Eschborn, Germany) with a pore size of 0.22 μm using a Millipore filtration tower, followed by washing with 1 × PBS and successively dehydrated with 50, 80, and 96% of ethanol. Samples were stored at −20°C until further analysis.

### 2.2 Microbial cell counts

Absolute bacterial counts in the water samples were quantified by catalyzed reporter deposition-fluorescence *in situ* hybridization (CARD-FISH) using the general probes EUB338 I-III (targeting most bacteria, including *Verrucomicrobia* and *Planctomycetes*) (Daims et al., 1999; Pernthaler et al., 2002). In addition, a NON338 (antisense EUB338) was used as our negative control. Briefly, cells on filters were coated with 0.2% low-melting point agarose, and bacteria were permeabilized with lysozyme (10 mg/mL in 0.05 M EDTA pH 8.0, 0.1 M Tris–HCl pH 7.5) and achromopeptidase (60 U/mL in 0.01 M NaCl, 0.01 M Tris–HCl). Cells were then incubated in 0.15% H_2_O_2_ in absolute methanol to inactivate the endogenous peroxidases. Horseradish peroxidase (HRP) labeled EUB338 I-III (working solution 50 ng/μL; Biomers, Ulm, Germany) in standard hybridization buffer (with 35% formamide; 1:300 vol/vol probe dilutions) (Pernthaler et al., 2002) were used to hybridize the 16S rRNA of bacteria at 46°C for 3 h. Subsequently, the filters were incubated for 15 min at 48°C in a prewarmed washing buffer. Following this, CARD was performed at 46°C for 20 min in the dark using a standard amplification buffer (Pernthaler et al., 2002) containing Alexa Fluor 594 labeled tyramides and thiomersal. Total cells were counterstained for 10 min with 1 μg/mL of 4′,6′-diamidino-2-phenylindole (DAPI). The cells were then embedded in a mixture of Citifluor and Vecta Shield (4:1 vol/vol) for fluorescence microscopy. Hybridizations by CARD-FISH were evaluated using fluorescence microscopy with an Axio Imager.Z2 microscope (Carl Zeiss, Germany) equipped with a 63×/1.40 Oil Ph 3 M27 Plan-Apochromat objective lens and filter sets for DAPI and Alexa Fluor 594. Images were captured by a monochromatic camera as black and white images, which were subsequently automatically colored and overlaid by Zen Pro software. For each filter, at least ten fields of view were acquired to count bacterial cells and total cells.

### 2.3 qPCR of antibiotic resistance genes

Total genomic DNA was extracted from biomass collected on filters using NucleoSpin^®^ Microbial DNA extraction kits (Macherey Nagel, Düren, Germany) following the manufacturer’s instructions. The target antibiotic resistance genes (ARGs) for quantification include two sulfonamide resistance genes (*sul1* and *sul2*), three tetracycline resistance genes (*tetA*, *tetM*, and *tetX*), and 16S rRNA genes as a surrogate for bacterial numbers. The absolute abundance of these genes was quantified by SYBR Green-based quantitative PCR (qPCR) with established primers and annealing temperatures (see Supplementary Table S1), using four technical replicates per sample. Calibration standards for *sul*1, *sul*2, and the 16S rRNA gene were the same as previously described (Haenelt et al., 2023b), while purified PCR amplicons generated from the sites were utilized for tet*A*, tet*M*, and tet*X*. The qPCR mixture contained 6.25 μL KAPA SYBR^®^ FAST (Sigma Aldrich, MO, United States), 4.75 μL ddH_2_O, 0.25 μL of forward and reverse primers (both at 10 μM), and 1 μL of template. Measurements were conducted on a StepOnePlus Real-Time PCR System with software version 2.1 (Applied Biosystems, MA, United States). The protocol was as follows: 95°C for 2 min, followed by 40 cycles of [95°C for 3 s, annealing (refer to Supplementary Table S1 for the annealing temperatures of each primer pair) for 20 s, and 72°C for 20 s], capped by a final step of extension for 3 s at 95°C. The relative abundances of ARGs were calculated as absolute abundance divided by the absolute abundance of the 16S rRNA gene. The detection limit for each gene was below five copies/μL reaction.

### 2.4 Metagenomic analysis

#### 2.4.1 Metagenomic sequencing

Metagenomic sequencing of the extracted DNA was performed on an Illumina NovaSeq 6,000 machine, as described before (Bacci, 2021). The extracted DNA was first sheared into 210–225 bp fragments, and then 100 ng was utilized to construct a paired-end library with a read length of 150 bp, following the manufacturer’s instructions with an Illumina DNA Prep Kit. A total of 73.3G, 90.6G, and 32G bases were generated for Site 1, Site 2, and Site 3, respectively.

#### 2.4.2 Read-based analysis

To recover 16S rRNA genes from the metagenomes, we used MATAM v1.6.0 (Pericard et al., 2018). First, paired-end reads were interleaved using the script reformat.sh from bbtools v37.62.^1^ Then, 16S rRNA genes were recovered using matam-assembly.py with the flag “--perform_taxonomic_assignment” for assigning taxonomies using SILVA 138.1 SSU Ref NR99 as reference. To remove chimeras, we used Vsearch v2.21.1 (Rognes et al., 2016) with the flag “--uchime_ref,” again referencing the SILVA database. Bacterial abundance was calculated using reads per kilobase per million reads (RPKM). To compare the calculated abundances across samples, we utilized MATAM’s script matam_compare_samples.py.

To identify ARGs, all clean sequencing reads were blasted against the Comprehensive Antibiotic Resistance Database (CARD, v3.1.4) (Alcock et al., 2020) with Diamond (v2.0.14, −query-cover 75, −id 90, −e-value 1e-5) as the first step to determine the antibiotic resistome. The list of ARGs was curated by re-analyzing the respective reads in CARD and via BLASTn in GenBank, together with literature searches. The relative abundance of ARGs was calculated according to an equation published previously (Zhang et al., 2022):

$$\begin{array}{l} \mathrm{Relative}\;\mathrm{abundance}=\\ \overset{n}{\underset{1}{\sum }}\frac{N_{\mathrm{target}\;\mathrm{gene}-\mathrm{like}\;\mathrm{sequence}}\times L_{\mathrm{reads}}/L_{\mathrm{reference}\;\mathrm{sequence}}}{N_{16S\;\mathrm{sequence}}\times L_{\mathrm{reads}}/L_{\mathrm{reference}\;\mathrm{sequence}}} \end{array}$$

where *N_target gene-like sequence_* is the number of reads mapped to the reference database; *L_reference sequence_* is the sequence length of the reference sequence; *L_reads_* is the read length (150 bp); *N_16S sequence_* is the number of clean reads classified as 16S rRNA; and *L_16S sequence_* is the average length of the 16S rRNA.

#### 2.4.3 Contig-based analysis

Each sample’s clean reads were individually assembled using MEGAHIT (v1.1.3, −mini-contig-len 1,000) (Li et al., 2016). The open reading frames (ORFs) were predicted using Prodigal v2.6.3 (−meta) and then searched against the database of CAR (−query-cover 70, −id 80, −e-value 1e-10). The contigs carrying ARGs (CCAs) were taxonomically classified with Taxator-tk (v1.3.3), identifying likely evolutionary neighbors from sequence similarities (Dröge et al., 2015) and CAT for homology searches on the ORF level (Von Meijenfeldt et al., 2019). Other ORFs on the CCAs were annotated using Bakta (Schwengers et al., 2021). Then, the list of CCAs was manually curated by blasting the ARG again in CARD and assessing the output for the following: (i) how much of the protein sequence in CARD was covered by the query sequence; (ii) the amino acid similarity to lower-scoring hits in CARD; and (iii) the presence of known point mutations in *Mycobacterium tuberculosis rpoB* ^2^ in the query *rpoB* genes, all of which had the *M. tuberculosis* gene as the closest homolog in CARD. In addition, SMART (Letunic et al., 2021) was used to explore the domain architecture of predicted efflux pump proteins encoded on the CCAs. The mobility of the DNA sources of the CCAs was evaluated threefold. First, the ORFs located on the CCAs were blasted against the ICEberg (integrative and conjugative elements) database (Bi et al., 2012). Second, PlasFlow v1.1 (Krawczyk et al., 2018) was used to assess whether a particular CCA may have been derived from a plasmid as indicated by its sequence signature, and third, by considering information in CARD on the mobility of the respective ARG and the inspection of the gene content of each contig supported by BLASTn of the entire CCA. The abundance of each CCA in the datasets was calculated as RPKM by mapping the clean reads with Bowtie2 (version 2.1.4) in the very-sensitive mode (-D 20 -R 3 -N 1 -L 20 -i S,1,0.50) (Langmead and Salzberg, 2012). In addition, individual ARGs were extracted from the CCAs, and their RPKMs were determined by mapping reads using Bowtie2 in the very-sensitive mode. Contigs were then aligned with reference sequences from GenBank using progressiveMauve (Darling et al., 2010).

#### 2.4.4 Generation and analysis of metagenome-assembled genomes

Metagenome-assembled genomes (MAGs) were constructed from the MEGAHIT output as follows: (1) by binning using metaBAT2 (Kang et al., 2019), MaxBin2 (Wu et al., 2016) and CONCOCT (Alneberg et al., 2013), respectively; (2) by refining the bins using the Bin_refinement module in the MetaWRAP (v1.3.0) (Uritskiy et al., 2018) with completeness >70% and contamination <5%; (3) and by combining and de-replicating the refined bins through dRep (Olm et al., 2017). The de-replicated MAGs were taxonomically classified by GTDB-Tk (v1.5.0) (Chaumeil et al., 2020). The abundances are expressed as genome copies per million reads (GPM) and were calculated with Salmon (installed in the Quant_bin module in MetaWrap) in a similar way to calculating transcripts per million in RNAseq analysis^3^ (Uritskiy et al., 2018). ARGs were searched for using CARD. All high-quality MAGs were annotated through the NCBI Prokaryotic Genome Annotation Pipeline. Annotation of one MAG (Site 3-bin.8, see Section 4.3) was carried out with Bakta prior to submission to GenBank. Whole genome alignment of MAG Site 3-bin.8 with genomes from GenBank was carried out with progressive Mauve. Average Nucleotide Identity (ANI) was computed with FastANI (Jain et al., 2018).

#### 2.4.5 Statistical analysis and generation of graphics

Bacterial and total cells were counted using Fiji (image processing package based on ImageJ). Heatmaps were plotted in R (v4.1.0)/Python (v3.9.2), bar charts were created in Excel, and Geneious Prime 2024^4^ was used to depict gene arrangement.

## 3 Results

### 3.1 Bacterial abundance and community composition at the sampling sites

**Figure 1:**
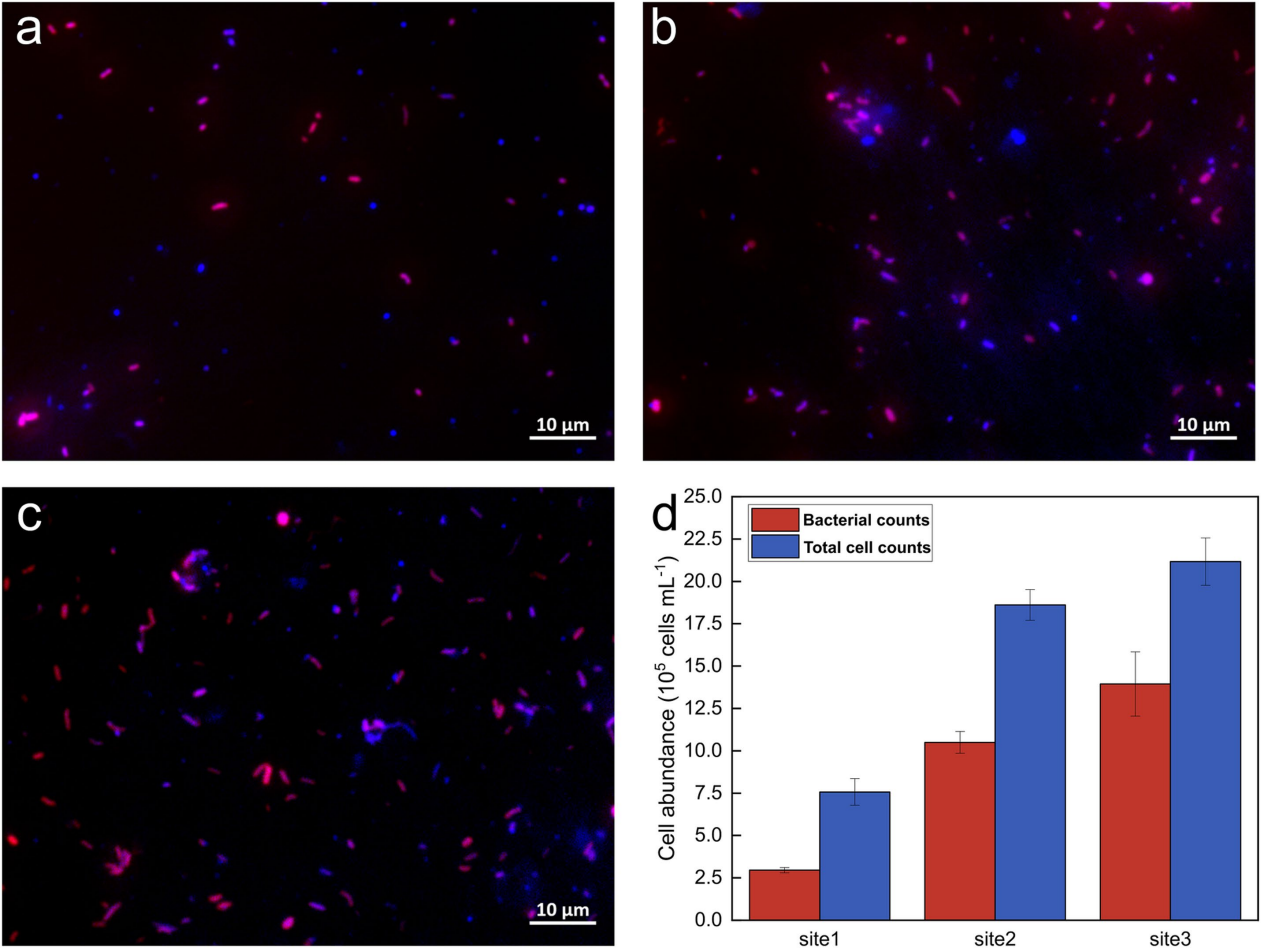
Representative fluorescence images and cell counts from CARD-FISH. **(A–C)** Overlay images of DAPI staining and the *16S rRNA*-targeting *EUBI-III* probe mix at Sites 1, 2, and 3, respectively; **(D)** Bacterial and total cell abundances at the sampling sites. Total cell abundances are shown in blue, while bacterial cells appear in pink, resulting from the overlay of the Alexa594 tyramide dye (red) and DAPI (blue) signals.

The abundance of bacterial and total cells was determined to assess bacterial abundance along the Holtemme River from a near-pristine location to sites downstream of two WWTPs and to enable comparisons with previous studies of microbial communities in the same river (Haenelt et al., 2023a,b). This was accomplished using CARD-FISH and DAPI staining techniques, along with qPCR quantification of 16S rRNA gene copy numbers. Based on the staining results (Figure 1), total cell counts were measured at 7.57 × 10^5^ ± 7.86 × 10^4^, 1.86 × 10^6^ ± 9.04 × 10^4^, and 2.12 × 10^6^ ± 1.40 × 10^5^ cells per milliliter of water at Site 1, Site 2, and Site 3, respectively. Among the total cell counts, 39.3 ± 3.7%, 56.5 ± 4%, and 65.7 ± 5.6% at Sites 1, 2, and 3, respectively, were identified as bacteria. The quantification of the 16S rRNA gene using qPCR aligned closely with the CARD-FISH results, demonstrating a similar trend in bacterial abundance. Specifically, about one log unit increased from the near-pristine site (Site 1) to Site 3 (Supplementary Figure S2c).

Metagenomic analysis was performed on deeply sequenced samples (32G – 90.6G bases per sample; 28.67, 34.80, and 12.01 Mio paired-end reads for Site 1, Site 2, and Site 3, respectively). 16S rRNA gene sequences were extracted from the metagenomic data and assembled to reveal the microbial community composition. In total, 1593, 3122, and 1091 16S rRNA gene scaffolds (>500 bp) were assembled from Site 1, Site 2, and Site 3, respectively. The proportion of bacterial sequences among the retrieved samples was 77.2, 76.0, and 71.9%, respectively (Supplementary Figure S3a). The remaining sequences were either unclassified scaffolds or *Archaea-related* sequences (data not shown). The bacterial 16S rRNA gene scaffolds across all samples were classified into 18 phyla. The dominant phylum was *Proteobacteria* (44.07%), followed by *Bacteroidetes* (5.91%), *Actinobacteria* (3.58%), and *Chlamydiae* (3.48%) (Supplementary Figure S3a). All these phyla, with the exception of *Chlamydiae*, are the most frequently detected phyla in urban river water (Tang et al., 2016; Abia et al., 2018; Wang et al., 2018). Among the top 50 genera, the genus *Rhodoferax* (10.13%) was predominant at all sampling sites, followed by *Flavobacterium* (3.26%). Sites 2 and 3 displayed similar microbial community compositions at the genus level and exhibited greater microbial diversity than Site 1 (Supplementary Figure S3b). The most abundant bacterial genera, *Aeromonas*, *Acinetobacter*, *Aliarcobacter*, *Mycobacterium*, *Pseudomonas*, *Romboutsia*, *Trichococcus*, and *Thiothrix,* have been frequently detected in effluents of WWTPs (Guo and Zhang, 2012; Ye and Zhang, 2013; Shi et al., 2023; Cheng et al., 2024). These genera exhibited higher relative abundance at Sites 2 and 3 (above 0.1%) compared to Site 1 (below 0.1% or not detected), with the exception of *Pseudomonas*, which showed similar abundance at Sites 1 (0.37%) and 2 (0.41%). Among these, *Aeromonas*, *Acinetobacter*, *Aliarcobacter*, *Mycobacterium*, and *Pseudomonas* are genera that contain pathogenic bacteria (Ye and Zhang, 2013; Chen X. et al., 2019; Chen H. et al., 2019; Yuan et al., 2021; Aoki et al., 2023). Bacterial genera of *Trichococcus* and *Thriothrix* contain species that relate to filamentous bulking in WWTPs (Howarth et al., 1999; Guo and Zhang, 2012). *Thriothrix* was detected only at Site 3, but with a high relative abundance of 2.5%, divided into three phylotypes (1.94, 0.35, and 0.2% relative abundance).

### 3.2 Antibiotic resistome along the Holtemme River

Five ARGs (*sul1*, *sul2*, *tetA*, *tetM,* and *tetX*) were selected as indicators of anthropogenic activities and quantified using qPCR (Supplementary Figure S2). Overall, Sites 2 and 3 had a higher absolute abundance of *sul1*, *sul2*, *tetA*, and *tetM* genes in comparison with those at Site 1. The highest absolute abundances of *sul1*, *sul2*, and *tetM* were observed at Site 2, with values of 1.2 × 10^5^, 5.5 × 10^4^, and 6.0 × 10^3^ copies per 100 mL of water, respectively. Site 3 followed with abundances of 1.0 × 10^5^, 4.7 × 10^4^, and 3.5 × 10^3^ copies per 100 mL of water for *sul1*, *sul2*, and *tetM*, respectively (Supplementary Figure S2c). The highest abundance of *tetX* was recorded at Site 1, reaching 4.9 × 10^3^ copies per 100 mL of water, approximately double the levels observed at Sites 2 and 3, thereby supporting the hypothesis of the origin of this gene in environmental bacteria (Yang et al., 2004).

#### 3.2.1 Read level analysis

**Figure 2:**
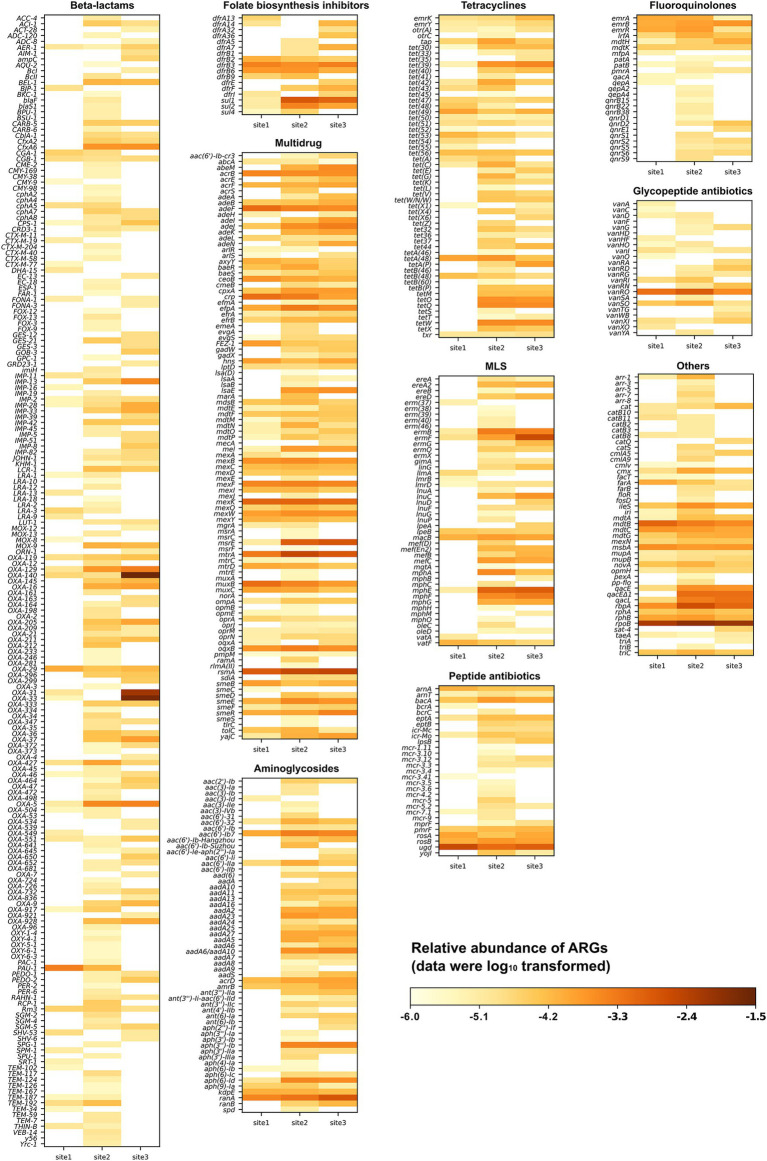
Relative abundance of various antimicrobial resistance gene types and subtypes detected at three sampling sites along the Holtemme River (site 1 is near-pristine, while sites 2 and 3 are influenced by effluent from wastewater treatment plants). The drug class referred to as “others” includes aminocoumarin, antibacterial free fatty acids, disinfectants, intercalating dyes, elfamycin, fosfomycin, mupirocin, nitroimidazole, nucleoside, phenicol, pleuromutilin, rifamycin, and triclosan.

In total, the reads from the three sites were mapped to 593 distinct ARGs spanning 22 drug classes. The proportions of shared and unique ARGs at the three sites are illustrated in Supplementary Figure S4 using a Venn diagram. A total of 226, 503, and 351 ARGs were identified at Site 1, Site 2, and Site 3, respectively. Of these, 25% were present at all three sites, 24.5% were found exclusively at Sites 2 and 3, and 6.1 and 10% were shared between Site 1 and either Site 2 or 3. These patterns highlight the differences in water quality between the near-pristine Site 1 and the affected Sites 2 and 3 exposed to WWTP effluent. Site 2 had the highest proportion of unique ARGs (29.3%), indicating regional variations in WWTP effluents. The abundance of all detected ARGs is shown in Figure 2. Among them, ARGs coding for resistance to sulfonamides and trimethoprim were categorized together as “folate biosynthesis inhibitors.” The drug class “others” includes aminocoumarin, antibacterial free fatty acids, disinfecting agents, intercalating dyes, elfamycin, fosfomycin, mupirocin, nitroimidazole, phenicol, pleuromutilin, rifamycin, and triclosan. The majority of the ARGs confer resistance to beta-lactams (32.6%), followed by those associated with multidrugs (16.9%), aminoglycosides (9.6%), tetracyclines (9.3%), and macrolides, lincosamides, and streptogramins (MLS; 7.9%) (Figure 2).

**Figure 3:**
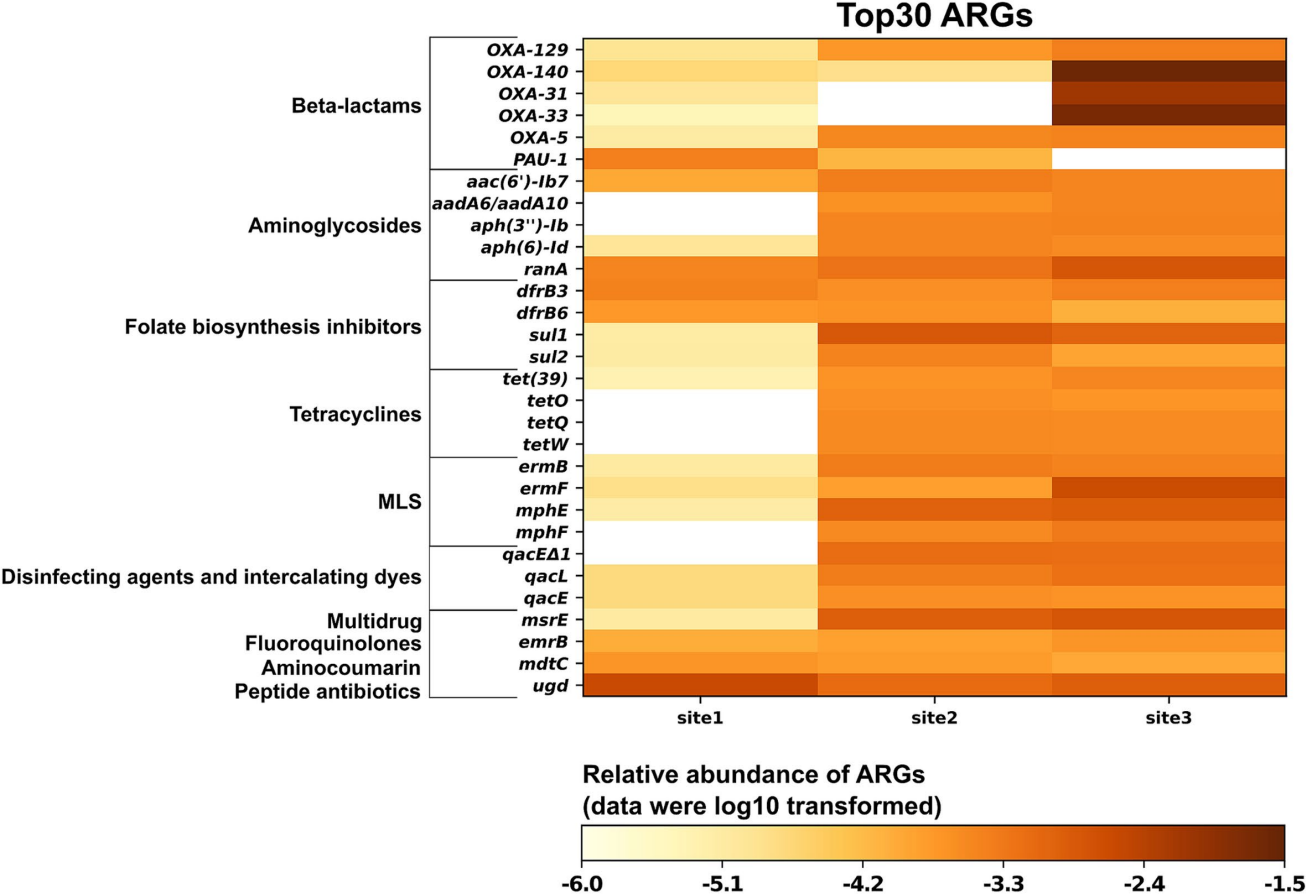
Relative abundance of the 30 most prevalent antimicrobial resistance genes from three sampling sites along the Holtemme River (site 1 is nearly pristine; sites 2 and 3 are both affected by the effluent from wastewater treatment plants).

Subsequently, we generated a curated list of the 30 most abundant ARGs based on read-level analysis, focusing on those with clinical or veterinary significance (Figure 3). The following genes conferring intrinsic resistance were excluded from this list, although they were abundant: (i) *bacA* (also known as *uppP*), which encodes an undecaprenyl pyrophosphate phosphatase involved in peptidoglycan biosynthesis and confers resistance to bacitracin in bacteria when overexpressed (El Ghachi et al., 2004). In Gram-negative bacteria such as the abundantly detected *Rhodoferax* (*Comamonadaceae*, Supplementary Figure S3), *bacA* / *uppP* is part of the standard chromosomal gene repertoire; (ii) nearly all ARGs encoding multidrug efflux pumps, as they confer clinically relevant levels of resistance in pathogenic bacteria only when overexpressed due to a promoter mutation or when present on a medium-high copy number plasmid (Atin et al., 2019). All reads were mapped within the respective efflux pump-encoding gene; therefore, the question of whether any of these genes were under the control of a mutated promoter or located on a plasmid could not be addressed. Furthermore, visual inspection of the read alignments with the significantly longer efflux pump genes raised questions about the validity of assigning the reads to a specific ARG, as the covered regions were almost always in a region conserved across multiple efflux pumps; (iii) all hits to *rpoB* were excluded, as none of them covered any of the mutations listed in CARD that confer resistance to rifampicin. The remaining 30 ARGs belong to 10 drug classes: beta-lactams, aminoglycosides, folate biosynthesis inhibitors, tetracyclines, disinfecting agents and intercalating dyes, multidrug, fluoroquinolones, MLS, aminocoumarin, and peptide antibiotics. A total of 23, 28, and 29 ARGs were detected at Site 1, Site 2, and Site 3, respectively. Most of these ARGs exhibited higher abundance at Site 2 and Site 3 (a total of 22 ARGs) compared to Site 1. The exceptions were *ugd*, *PAU-1,* and *mdtC*, which confer resistance through antibiotic target alteration, antibiotic inactivation, and antibiotic efflux, respectively. Most ARGs encoding resistance to oxacillin-hydrolyzing type extended-spectrum-*β*-lactamases (*bla*_OXA_ or *OXA* for short) and MLS displayed the highest abundance at Site 3, while those conferring resistance to folate biosynthesis inhibitors and tetracyclines showed the highest relative abundance at Site 2. The relative abundances of *sul1* and *sul2* exhibited a pattern similar to that detected by qPCR across all sites, with the highest abundances observed at Site 2 and the lowest at Site 1 (Figure 2; Supplementary Figure S3a). Additionally, the abundances of *sul1* and *sul2* were approximately 1–2 orders of magnitude higher than those of *tet* genes (*tetA*, *tetM,* and *tetX*) at sites 2 and 3, consistent with the qPCR results (Supplementary Table S2). Among the analyzed ARGs, *OXA-140*, *OXA-33*, and *OXA-31* exhibited higher relative abundance at Site 3 compared to sites 1 and 2. The relative abundances at Site 3 were 2.56 × 10^−2^, 1.92 × 10^−2^, and 7.12 × 10^−3^ copies per 16S rRNA for *OXA-140*, *OXA-33*, and *OXA-31*, respectively, representing 3–4 orders of magnitude greater than those detected at sites 1 and 2 (Figure 3).

The five highly abundant *OXA* genes belong to two families: the *OXA-1* family (*OXA-31*, *OXA-33*, and *OXA-140*) and the *OXA-5* family (*OXA-5* and *OXA-129*). The pairwise nucleotide similarities among members within each family are high, ranging from 99.72 to 100% (at varying gene lengths) for the *OXA-1* family and 94.2% for the *OXA-5* family. Furthermore, *OXA-33* and *OXA-140* are identical to *OXA-4* (see Section 4.2.2), but they have unique gene numbers because of differences in length (*OXA-4* is 831 bp, while *OXA-140* is 705 bp and *OXA-33* is 768 bp). Re-analyzing the reads assigned during CARD analysis to a specific *OXA-1* family gene showed that 40 to 100% of these reads mapped equally well to another gene in this family (e.g., *OXA-4*) (Supplementary Figure S5). While we present the top hit from the initial CARD analysis in Figure 3, we note that relying solely on metagenomic reads does not always allow for differentiation between closely related *OXA* genes.

#### 3.2.2 Contig level analysis

**Figure 4:**
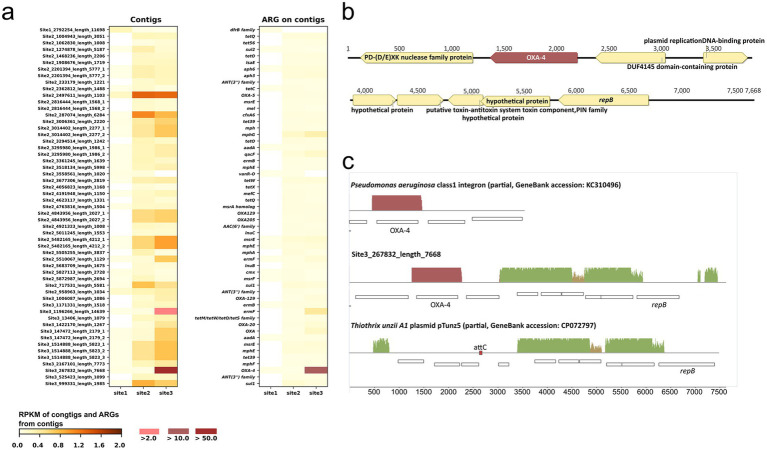
Contigs and antibiotic resistance genes (ARGs) associated with each contig. **(A)** Reads per kilobase per million reads (RPKM) of contigs and ARGs indicate that the ARGs in each row of the “ARG on contigs” heatmap are found on the corresponding contigs in the heatmap on the left. The suffixes “_1”, “_2”, and “_3” in some contig names denote those contigs that contain multiple ARGs; **(B)** gene arrangement on contig Site 3_267832_length_7668; **(C)** alignment of contig Site 3_267832_length_7668 with GenBank sequences (the size of pTunz5 in the alignment is 7.9 kb, which is half the size of the plasmid in GenBank).

An initial analysis using CARD identified a total of 277 contigs with ARGs assembled from the metagenomic data across all sampling sites. The list of contigs was manually curated using information from CARD and the content of each contig. Similar to the read-level analysis, contigs with *bacA*, *rpoB,* and most genes coding for multidrug efflux pumps were removed, leaving 46 contigs for further analysis (Figure 4A). Reads from Site 1, Site 2, and Site 3 mapped to 27, 46, and 45 contigs, respectively. The human health risk of the ARGs, according to a recent categorization, is presented in Supplementary Table S3 (Zhang et al., 2021).

Eight contigs contain more than one ARG: Site 2_2201394_length_5777 [*aph(3″)-Ib*, *aph(6)-Id*], Site 2_2816444_length_1568 (*msrE*, *mel*), Site 2_3014402_length_2277 (*mph*, *mphG*), Site 2_3295980_length_1986 (*aadA6*, *qacF*), Site 2_4843956_length_2027 (*OXA-129*, *OXA-205*), Site 2_5482165_length_4212 (*msrE*, *mphE*), Site 3_147472_length_2179 (*OXA*, *aadA6*), and Site 3_1514888_length_5823 (*msrE*, *mphE*, *tet39*). For 41 out of the 46 contigs, evidence from PlasFlow prediction, ICEberg analysis, or the closest GenBank hit indicated that they were derived from or associated with mobile genetic elements (Supplementary Table S3). Only one contig (Site 1_2792254_length_11698) displayed a higher RPKM value at Site 1 than at the other two sites. That contig carried a *dfrB* family gene, predicted to code for a dihydrofolate reductase that confers resistance to trimethoprim. Throughout the list of contigs, RPKM values for the entire contig and its extracted ARG(s) were not always consistent; some contigs showed proportionally higher RPKM values than the corresponding ARG (e.g., Site 2_2497611_length_1103 with *OXA-5*), suggesting that the samples also contained corresponding DNA fragments without the ARG.

The most abundant contig was Site 3_267832_length_7668 at Site 3, which carried an *OXA-4* gene, as described in detail in the next paragraph. Five additional contigs contain *OXA* genes: *OXA-5*, *OXA-205,* and *OXA-129* together on one contig; *OXA-129* on another contig; *OXA-20*; and an *OXA* gene of uncertain family affiliation. As anticipated from the read-based analysis, the RPKM values of these genes followed the order Site 3 > Site 2 > > Site 1. Similarly, as predicted from the read-based analysis, the RPKM of *sul1* and *qacEΔ1*, which are part of class 1 integron, followed the order Site 2 > Site 3 > > Site 1 (Supplementary Table S3).

Contig Site 3_267832_length_7668 (Figure 4B) contains an *OXA-4* gene cassette of 1 kb spanning the R’ sequence of *attC* for the class 1 integron integrase (Ghaly et al., 2021) upstream to a full *attC* site downstream of *OXA-4*. In a BLASTn query, we found 48 identical homologs of the *OXA-4* cassette, with 44 of them in *Enterobacteriaceae* and *Pseudomonas aeruginosa*. Across all hosts, the cassette was almost always part of a class 1 integron. The other genes in the contig are predicted to code for RepB, responsible for recognizing and processing a plasmid’s origin of replication (Boer et al., 2009), a hypothetical protein, a PIN toxin-antitoxin system, a plasmid-replication DNA-binding protein, and two nucleic acid-interacting proteins, specifically a (PD-(D/E)) XK nuclease superfamily protein and a DUF4145 domain-containing protein. All but the latter two proteins and *OXA-4* have closest homologs in *Thiothrix* spp. and other *Thiotrichaceae* (67–99.3% aa identity), mostly encoded on small, unclassified plasmids. Figure 4C presents an alignment of contig Site 3_267832_length_7668 with half of the closest plasmid homolog, pTunz5 from *Thiothrix unzii* strain A1 (GenBank accession CP072797), and a class 1 integron fragment containing *OXA-4* (accession KC310496). Only half of the 14.8 kb pTunz5 plasmid was used for the alignment, as both halves of the plasmid assembly are identical. The contig shares 97% nucleotide similarity over 40% coverage with the pTunz5 half. Notably, each pTunz5 half contains an *attC* site (RYYYAAC – 121 nt – GTTRRRY) at a position corresponding to *OXA-4* in contig Site 3_267832_length_7668. Furthermore, the first and last 141 bp of Site 3_267832_length_7668 are perfect repeats, which would facilitate the circularization of this contig. Therefore, it is likely that contig Site 3_267832_length_7668 is derived from a pTunz5-like plasmid where an *OXA-4* gene cassette was inserted.

#### 3.2.3 MAG level analysis

Metagenomic assembly and binning were conducted to examine the co-occurrence of ARGs in the recovered MAGs. A total of 46 dereplicated high-quality MAGs were obtained from all samples, which were taxonomically categorized into 10 phyla: *Proteobacteria* (16), *Actinobacteriota* (13), *Bacteroidota* (6), *Patescibacteria* (3), *Planctomycetota* (2), *Chlamydiota* (2), *Verrucomicrobiota* (1), *Bdellovibrionota* (1), *Chloroflexota* (1), and *Cyanobacteria* (1) (Supplementary Table S4). The most prevalent MAGs were from the *Proteobacteria* phylum (with abundances at Site 1, Site 2, and Site 3 measuring 1860.84, 1217.68, and 2317.362 GPM, respectively), followed by *Bacteroidota* and *Actinobacteriota*. This observation was consistent with the microbial composition identified through 16S rRNA gene analysis.

A total of nine out of the 46 MAGs carried ARGs, as identified by CARD analysis, most of which confer multidrug resistance such as *rsmA*, *msbA*, *cpxA*, *abeM*, *adeK*, *adeJ,* and *mtrA*. All multidrug resistance genes were on contigs of likely chromosomal origin, and none of them are considered high risk for human health (Zhang et al., 2021). Of the nine MAGs, one was retrieved from Site 1, six from Site 2, and two from Site 3. Five of the nine MAGs were assigned to bacterial genera commonly associated with WWTPs, and their abundance patterns were consistent with those observed at the read level (Supplementary Figure S3b). These included Site 2-bin.48, classified as *Acinetobacter* (with GPM values of 0.6, 23.1, and 60.6 at sites 1, 2, and 3, respectively), and three MAGs —Site 2-bin.11, Site 2-bin.20, and Site 2-bin.25 — classified as *Mycobacterium* (with total GPM values of 10.9, 158.5, and 68.0 at sites 1, 2, and 3, respectively). The MAG with the highest GPM abundance by far was Site 3-bin.8, recovered from Site 3 (GPM at Site 3 of 860.1; at Site 1 of 2.0, and at Site 2 of 1.0) (Supplementary Figure S3c). GTDB-Tk analysis affiliated this MAG with the genus *Thiolinea* but without species-level assignment. Further comparison using FastANI and Mauve of this MAG with genomes and MAGs of *Thiolinea* spp. available in GenBank revealed *Thiolinea eikelboomii* (GTDB taxonomy; *Thiothrix eikelboomii* in the List of Prokaryotic Names with Standing in Nomenclature, LPNS) (GCF_900167255.1) and a MAG of an uncultured *Thiolinea* sp. (GCF_937876535.1) recovered from a WWTP in the Charlotte area, NC, United States, as closest homologs. ANI values were 80.91 and 80.6% between MAG Site 3-bin.8 and the two closest homologs. ANI values with all other sequenced *Thiolinea* genomes were < 80%. Whole genome alignments revealed substantial synteny between MAG Site 3-bin.8, *T. eikelboomii*, and the MAG from the Charlotte area WWTP (Supplementary Figure S6), with an alignment of MAG Site 3-bin.8 with the genome of *Thiolinea disciformis* (GCF_000371925.1) shown for comparison. As the genome alignment indicated, MAG Site 3-bin.8 has a high completeness level of 98% and a low contamination level of 0.5%. The gene content of the MAG appears typical for the genus *Thiolinea* (Ravin et al., 2022). The three *Thiotrichaceae* phylotypes identified via 16S rRNA (Section 4.1) exhibited 98 to 99.3% nucleotide identity to the 16S rRNA gene sequence of *T. eikelboomii*.

## 4 Discussion

The Holtemme River has been extensively used as a model for investigating anthropogenic impacts on small rivers. In two recent studies, we mapped the abundance of the AMR indicator genes, *sul1*, *sul2,* and *intI1*, and of ARG cassettes within class 1 integrons along the anthropogenic gradient from a near-pristine site to downstream of the first WWTP at this river (Haenelt et al., 2023a,b). During the sampling periods, one of which was about 2 months after the present study, WWTP effluent was the principal source of these genes in the river. In this study, we further defined the impact of anthropogenic activities on the resistome of the Holtemme River using metagenomic sequencing, CARD-FISH, and qPCR. Bacterial abundance at the effluent-impacted sites 2 and 3 was approximately an order of magnitude higher than that at the near-pristine Site 1, consistent with previous abundance profiling (Kamjunke et al., 2019) and correlating with correspondingly higher absolute abundance of detected ARGs. Metagenomics revealed marked differences in the ARG profiles between the three sites. While it was anticipated that the ARG profile at the near-pristine site would differ from the two WWTP-impacted sites, the extent of the differences between the latter two sites, which are only about 20 km apart, was not expected. Site 2 exhibited a higher abundance of ARGs associated with resistance to sulfonamides and tetracyclines, whereas Site 3 showed a greater prevalence of ARGs conferring resistance to beta-lactams and MLS. Furthermore, the abundance of the class 1 integron, its presence inferred from the almost equal prevalence of *sul1* and *qacE*Δ*1*, which together are part of the 3′ conserved segment of that integron (Nardelli et al., 2012), appeared to be higher at Site 2 than at Site 3. These variations between Sites 2 and 3 could be attributable to differences in the composition of the wastewater discharged from the two WWTPs, demonstrating that the distribution of ARGs can show regional differences within the same watershed (Wang et al., 2020; Raza et al., 2021). The findings suggest that different indicator gene sets should be employed if regular monitoring of ARG pollution along the river is conducted using the qPCR method.

The beta-lactam resistance genes at Sites 2 and 3 were mainly of the *OXA* type, encoding oxacillinases (class D beta-lactamases) with strong hydrolytic activity against the semisynthetic penicillin oxacillin (Evans and Amyes, 2014). These genes are widely distributed among the ESKAPE pathogens (*Enterococcus faecium*, *Staphylococcus aureus*, *Klebsiella pneumoniae*, *Acinetobacter baumannii*, *Pseudomonas aeruginosa*, and *Enterobacter* species), which are of global concern due to their involvement in severe nosocomial infections (Pandey et al., 2021; Avci et al., 2023). Analysis of ARGs at the contig level indicated that all *OXA*-type genes found at Site 3 resided in mobile genetic elements, with half of these being linked to ESKAPE pathogens. This suggests that greater attention should be paid to the prevalence of these genes and their association with hosts at this site.

The *OXA-4* gene was by far the most abundant of all ARGs detected at the three sites. According to omics-based categorization, it is considered a “high risk” ARG (Supplementary Table S3) (Zhang et al., 2021). Here, the gene was found to be mostly associated with a plasmid-like DNA fragment based on RPKM of ARG and contig analysis. This DNA fragment was homologous to a group of small plasmids found so far only in members of the *Thiotrichaceae*, with plasmid pTunz5 from *T. unzii* strain A1 as the closest homolog in GenBank. Sequence evidence suggested that the *OXA-4* gene was inserted as a cassette into a pTunz5-like plasmid, with a class 1 integron in a member of the *Enterobacteriaceae* or *Pseudomonas aeruginosa* as a potential cassette donor. The high abundance of the pTunz5-like sequence was reflected by the high relative abundances of 16S rRNA gene sequences affiliated with *Thiotrichaceae*, which accounted for about 2.5% of all 16S rRNA gene sequences in the metagenome data set from site 3. Furthermore, a high-quality, high relative abundance MAG was recovered from Site 3 that was classified as *Thiolinea* sp. by GTDB-Tk analysis and ANI, with the genome of *T. eikelboomii* and a MAG from an uncultured *Thiolinea* sp. as the closest homologs. Although we cannot be certain about the actual evolutionary history of the *OXA-4* gene and the identity of the in-situ host at site 3, it is conceivable that it was harbored by a close relative of *T. eikelboomii* in a small plasmid. This possibility is significant because these filamentous microorganisms are common inhabitants in WWTPs but are also frequently found in rivers, streams, and other freshwater systems (Howarth et al., 1999; Boden and Scott, 2018; Ravin et al., 2022; Gureeva et al., 2024). Therefore, the present finding appears to be a case where a member of the *Thiotrichaceae* was an effective shuttle system for a clinically relevant ARG into the environment. Members of this family can cause WWTP malfunction by bulking (Howarth et al., 1999; Guo and Zhang, 2012), with *T. eikelboomii* being a notorious example (Williams and Unz, 1985). During bulking, filamentous bacteria like *T. eikelboomii* do not settle effectively in the clarifier stage of a WWTP, resulting in increased loads of organic matter and bacteria in the effluent. It is concerning if some of the bacteria leaving a WWTP carry clinically relevant ARGs and possess the ability to survive in the downstream water body. Understanding how these ARGs and ARBs spread through interconnected One Health compartments is crucial for developing effective strategies to combat the spread of AMR.

In this study, we revealed the effects of human activities on the microbial community and resistome in the Holtemme River, with the primary influence attributed to WWTP discharges, as in our previous study (Haenelt et al., 2023a,b). In addition, the in-depth analysis of metagenomic sequencing data revealed the likely horizontal transfer route of a highly abundant and clinically relevant ARG, *OXA-4*, with evidence showing its likely host being a filamentous bacterium, *T. eikelboomii*, that has been found in WWTPs worldwide. Metagenomic sequencing not only offered a comprehensive overview of microbial communities and the resistome in this river water but also provided information for the prediction of the transfer route of ARGs of clinical importance between organisms. To verify the prediction from metagenomic sequencing data, analytical methods that can link an ARG with phylogenetic markers could be applied (Moraru et al., 2010; Neufeld, 2017; Stalder et al., 2019; Diebold et al., 2021; Zeugner et al., 2021; Knecht et al., 2023).

## Data Availability

The datasets presented in this study can be found in online repositories. The names of the repository/repositories and accession number(s) can be found at: https://www.ncbi.nlm.nih.gov/bioproject/PRJNA888038/ and https://zenodo.org/records/14609579.

## Footnotes

^1^ https://sourceforge.net/projects/bbmap/

^2^ https://card.mcmaster.ca/ontology/39867

^3^ https://github.com/bxlab/metaWRAP/blob/master/Usage_tutorial.md

^4^ https://www.geneious.com
