# Supplementary material for: Riverine antibiotic resistome along an anthropogenic gradient
Source: Front Microbiol. 2025 Feb 26;16:1516033. doi: 10.3389/fmicb.2025.1516033 (PMC11897494; doi:10.3389/fmicb.2025.1516033)
Supplement: Supplementary file 1 [file Data_Sheet_1.docx]

Supplementary Material

Riverine antibiotic resistome along a gradient of anthropogenic impact

Gangan Wang^1^, Sarah Haenelt^1^, Felipe Borim Corrêa^2^, Ulisses Nunes da Rocha^2^, Florin Musat^1,3^, Junya Zhang^1,5*^, Jochen A. Müller^4*^**^ǂ^**, Niculina Musat^1,3^ **^ǂ^**

^1^Department of Technical Biogeochemistry, Helmholtz Centre for Environmental Research, Leipzig, Germany

^2^Department of Environmental Microbiology, Helmholtz Centre for Environmental Research, Leipzig, Germany

^3^Department of Biology, Section for Microbiology, Aarhus University, Aarhus, Denmark

^4^Karlsruhe Institute of Technology, Institute for Biological Interfaces (IBG 5), Eggenstein-Leopoldshafen, Germany

^5^State Key Joint Laboratory of Environmental Simulation and Pollution Control, Research Center for Eco-Environmental Sciences, Chinese Academy of Sciences, Beijing, China

ǂ contributed equally

^*^corresponding authors

niculina.musat@bio.au.dk

# jochen.mueller@kit.edu

**Figures:**

**Fig. S1** Location of the three sampling sites in the catchment of the Holtemme river in the Harz mountain region, Germany

**Fig. S2** Abundance of ARGs by qPCR

**Fig. S3** Microbial community composition by MATAM (based on 16S rRNA sequences extracted from metagenomic data

**Fig. S4** Venn diagram showing the number of site-specific and shared ARGs among sampling sites.

**Fig. S5** Percentage of reads mapped equally well to OXA-4 through blast.

**Fig. S6** Whole genome alignments of MAG Site3-bin.8 with MAG/genomes of an uncultured Thiolinea sp937873765 (GCF_937876535.1), Thiothrix eikelboomii (GCF_900167255.1) and Thiolinea disciformis (GCF_000371925.1) using Mauve.

**Tables:**

**Table S1.** Target genes, primer sequences, amplicon sizes and annealing temperature for qPCR assays

**Table S2.** The relative abundance of *sul1*, *sul2*, *tetA*, *tetM* and *tetX* detected by qPCR and metagenomic sequencing

**Tables S3** and **S4** are separate Excel files.


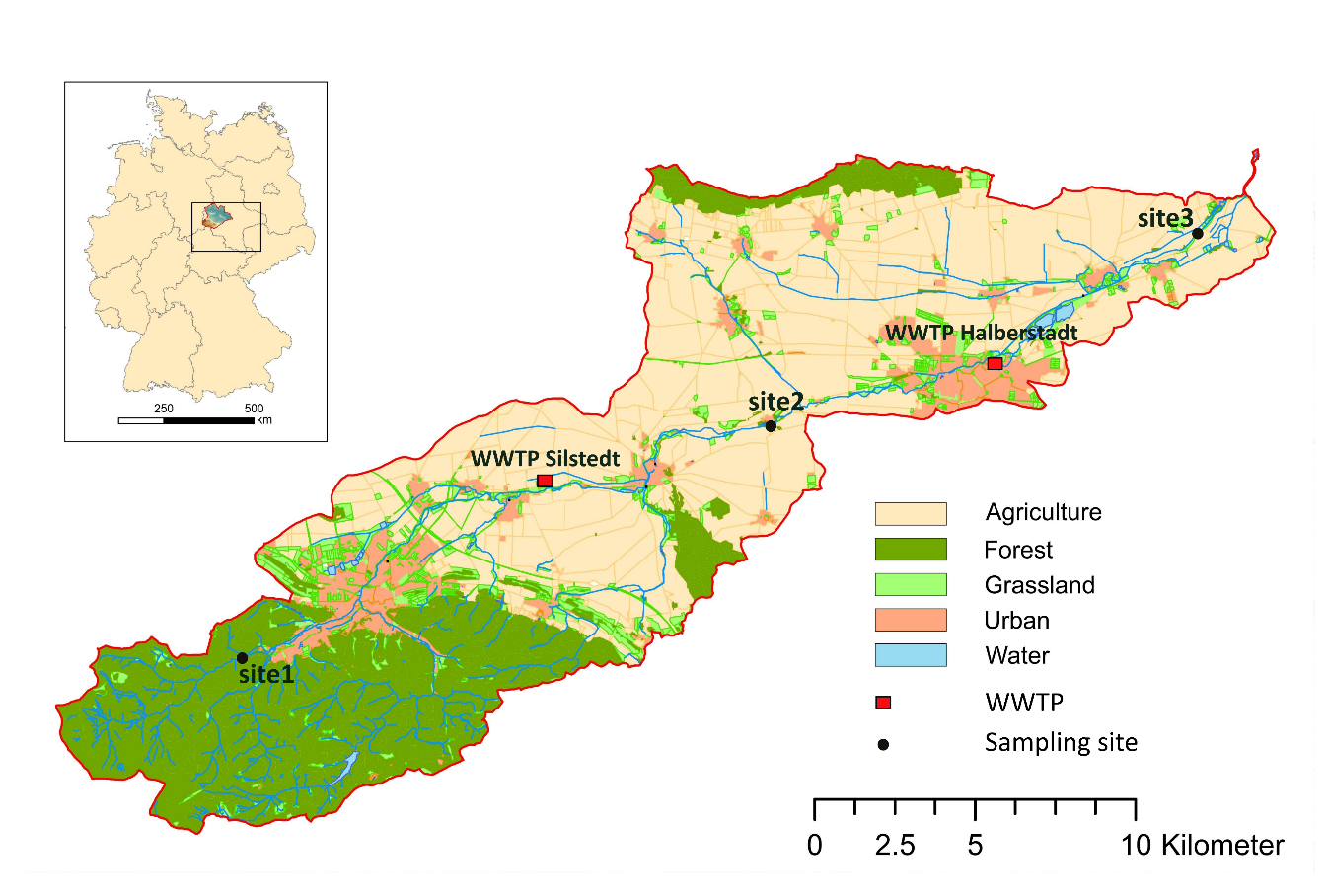


**Fig. S1** Location of the three sampling sites in the catchment of the Holtemme river in the Harz mountain region, Germany. Map created from Authorative Topographic–Cartographic Information System (ATKIS).


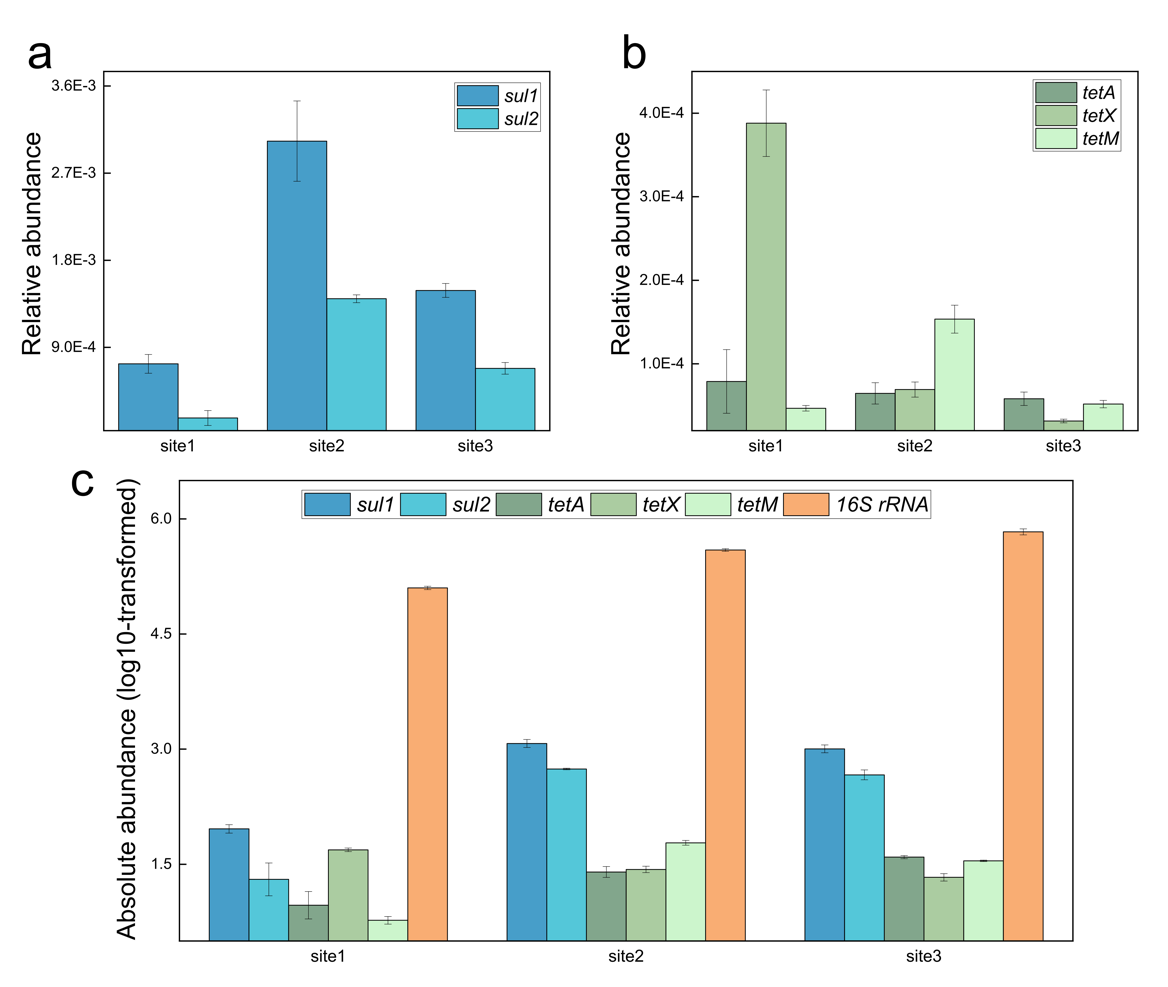


**Fig. S2** Abundance of ARGs determined by qPCR. a) Relative abundance of *sul* genes (ARG copies/16S rRNA gene copy number), b) Relative abundance of *tet* genes (ARG copies/16S rRNA gene copy number), c) Absolute abundance of 16S rRNA and all target ARGs (log10-transformed, unit: copies/100 mL water)


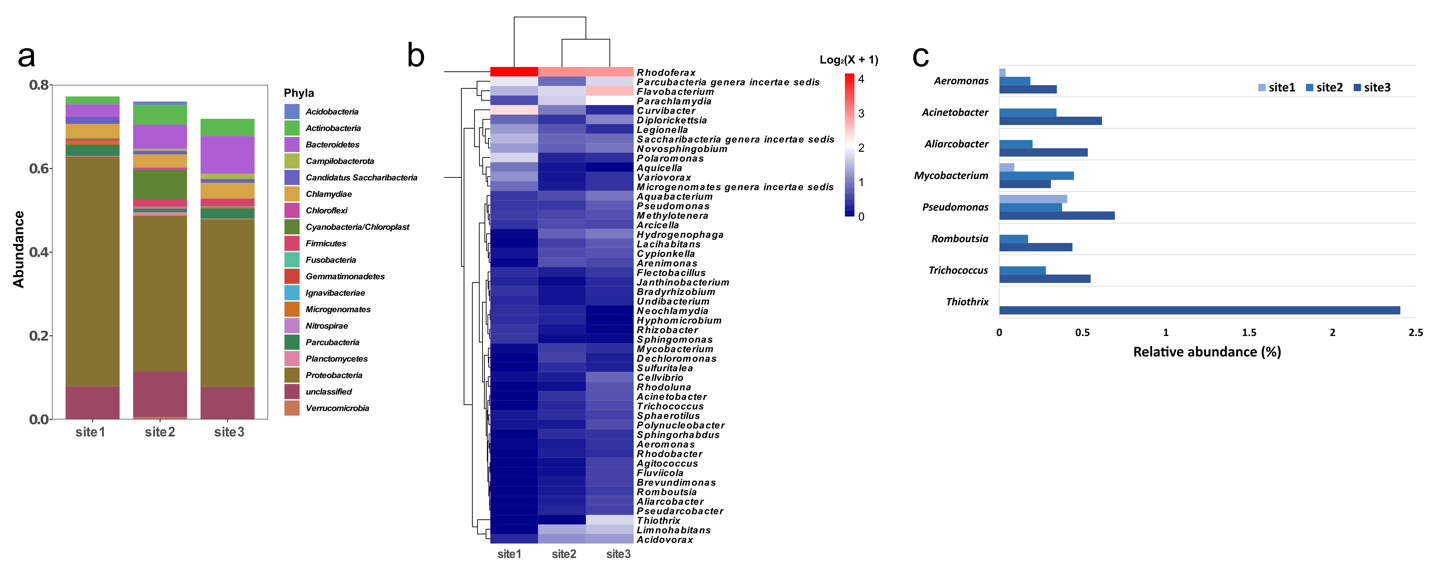


**Fig. S3** Microbial community composition by MATAM (based on 16S rRNA sequences extracted from metagenomic data). a) Relative abundance of bacteria at phylum level; b) Relative abundance of top50 bacteria at genus level (data were log_2_(x + 1) transformed, where x represents the relative abundance of each genus in %); c) The relative abundance of bacterial genera related with WWTPs.


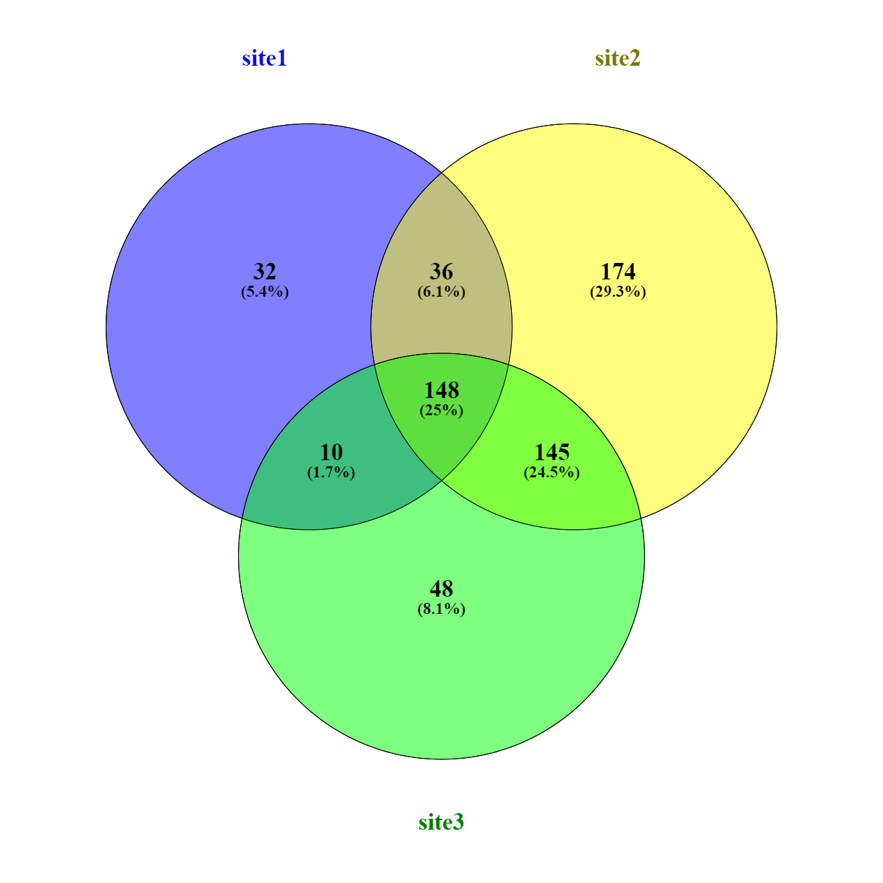


**Fig. S4** Venn diagram showing the number of site-specific and shared ARGs among sampling sites.


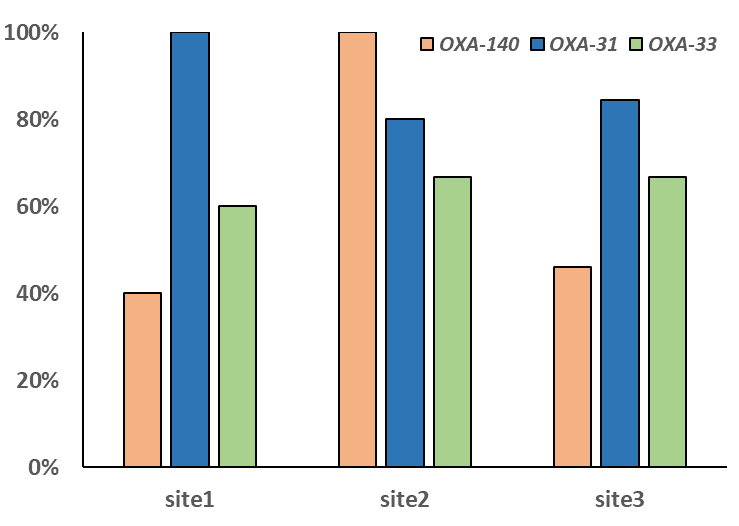


**Fig. S5** Percentage of reads obtained in this study mapping equally well to *OXA-4*.


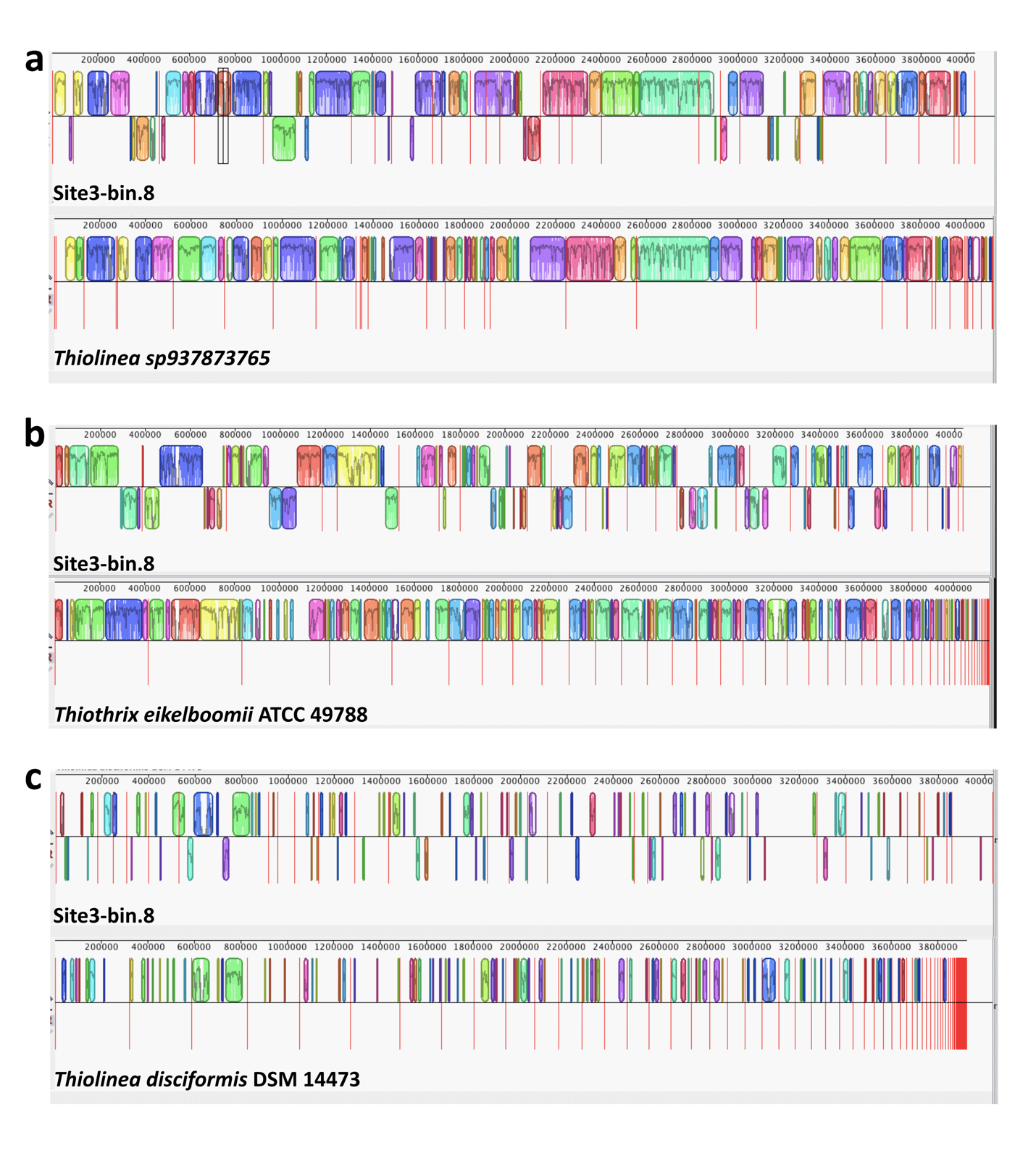


**Fig. S6** Whole genome alignments of MAG Site3-bin.8 with MAG/genomes of a) an uncultured *Thiolinea sp937873765* (GCF_937876535.1); b)*Thiothrix eikelboomii* (GCF_900167255.1) and c) *Thiolinea disciformis* (GCF_000371925.1) using Mauve.

**Table S1. Target genes, primer sequences, amplicon sizes and annealing temperature for qPCR assays**

| Target gene | Sequence (5’→3’) | Size (bp) | Annealing temperature (℃) | References |
| --- | --- | --- | --- | --- |
| *16S rRNA* | F: CCAGCAGCCGCGGTAATAC  R: CCGTCAATTCCTTTRAGTTT | 410 | 60 | (Wang et al., 2014) |
| *sul*1 | F: TGTCGAACCTTCAAAAGCTG  R: TGGACCCAGATCCTTTACAG | 113 | 60 |  |
| *sul*2 | F: ATCTGCCAAACTCGTCGTTA  R: CAATGTGATCCATGATGTCG | 89 | 60 |  |
| *tet*A | F: GCTACATCCTGCTTGCCTTC  R: CATAGATCGCCGTGAAGAGG | 210 | 60.9 | (Shin et al., 2020) |
| *tet*M | F: ACAGAAAGCTTATTATATAAC  R: TGGCGTGTCTATGATGTTCAC | 171 | 45 |  |
| *tet*X | F: GGACCCGTTGGACTGACTATGC  R: TACACCCATTGGTAAGGCTAAGT | 195 | 61 |  |

**Table S2. The relative abundance of *sul1*, *sul2*, *tetA*, *tetM* and *tetX* detected by qPCR and metagenomic sequencing**

| ARG | qPCR | | | metagenomic sequencing | | |
| --- | --- | --- | --- | --- | --- | --- |
|  | site1 | site2 | site3 | site1 | site2 | site3 |
| *sul1* | 7.30E-04 | 3.03E-03 | 1.49E-03 | 7.41E-06 | 1.48E-03 | 9.84E-04 |
| *sul2* | 1.71E-04 | 1.40E-03 | 6.84E-04 | 7.63E-06 | 3.64E-04 | 1.28E-04 |
| *tet(A)* | 7.89E-05 | 6.46E-05 | 5.82E-05 | 1.71E-05 | 3.24E-05 | 0 |
| *tetM* | 4.68E-05 | 1.53E-04 | 5.18E-05 | 0 | 5.51E-05 | 5.78E-05 |
| *tetX* | 3.88E-04 | 6.92E-05 | 3.16E-05 | 0 | 5.53E-05 | 3.57E-05 |
